# Training primary care staff in delivering the primary care consultation remotely: a systematic review

**DOI:** 10.3399/BJGPO.2023.0110

**Published:** 2023-11-29

**Authors:** Jo Parsons, Bilal Salman, Helen Leach, Eleanor Watson, Helen Atherton

**Affiliations:** 1 Unit of Academic Primary Care, Warwick Medical School, Coventry, UK

**Keywords:** education, medical, graduate, internship and residency, primary health care, remote consultation, self efficacy, staff development, general practitioners

## Abstract

**Background:**

Remote consultation is widely used in primary care, and its use has increased greatly since the onset of the COVID-19 pandemic. Despite this, primary care clinicians lack formal training in delivering remote consultation. There is a need to understand how training might best be delivered, and what evidence there is to support this.

**Aim:**

To summarise existing published literature about training primary care staff in conducting primary care consultation remotely, to outline which models of training may be effective, and to identify unanswered questions for future research.

**Design & setting:**

Systematic review of English language studies in primary care included in Medline (Ovid), Cochrane Database, PubMed, Embase (Ovid), Web of Science, and CINAHL from 2010–2021; and in Google results from 2010–2022.

**Method:**

Databases were searched using a predefined search strategy. Title, abstract, and full-text screening was conducted to identify eligible studies for inclusion in the review. The quality of included studies was assessed, and findings were synthesised to answer the research questions.

**Results:**

We included 10 studies. Seven examined training on remote consultation with trainee GPs or residents, and three examined training on remote consultation with qualified primary care clinicians. Training described led to positive change overall, including increased confidence and self-efficacy in delivering remote consultations. Furthermore, trainees reported increased use of remote consultation, increased efficiency, and increased engagement from patients. Studies where training involved workshops or didactic learning alongside experiential learning resulted in more positive feelings and more confidence about how technology could aid consultations.

**Conclusion:**

There is limited evidence on training primary care staff in conducting remote consultation. Available evidence indicates that training has a positive impact on the ability of clinicians and staff to deliver remote consultation.

## How this fits in

Remote consultation is widely used in primary care, and its use has increased greatly since the onset of the COVID-19 pandemic. This review synthesises the little evidence that currently exists on how to train primary care staff to conduct remote consultation, noting that what evidence can be found varies in method and application. It does, however, show that training can have a positive impact on clinicians and staff, including improved confidence and efficiency. This review highlights that further evidence is needed on how to train primary care staff in delivering remote consultation.

## Introduction

Telephone consultation is an established means of delivering consultations to patients in primary care settings.^1,2^ Its use has increased dramatically since the onset of the COVID-19 pandemic,^3^ making appropriate training in remote consulting vital to ensure adequate patient care is delivered. More recently, primary care has also used other remote consulting mediums (including video and online written consultation).^4–7^

Primary care staff are required to organise and deliver remote consultation, and while written support has been developed,^8,9^ a lack of formal training has been identified as a barrier to successful implementation and use of remote patient consultation.^4,10,11^

Within postgraduate medical training, consultation skills education has focused on face-to-face consulting.^12,13^ By contrast, medical schools are increasingly training students in using remote consultation.^14^ Primary care clinicians are expected to train medical students in delivering remote consultation^15^ despite not necessarily being supported to develop these skills. There is likely a knowledge gap for the cohort of primary care clinicians currently training and practising. Some steps have been made to integrate remote consulting into more specialised training, including supervised clinics, training days, and standardised guidance.^16,17^ Work on this integration is limited, however, and more understanding of primary care clinician training is needed.

Local, national, and international guidance on remote consulting has emerged from the COVID-19 pandemic.^8,9,18^ Although this guidance can assist primary care clinicians, it is not a substitute for official training, and may not be evidence-based. As the demand for staff training in primary care increases, we conducted a thorough analysis of empirical research. The objective of this review was to provide an overview of effective training models for primary care staff in conducting remote consultation, and to highlight areas that require further research.

## Method

This systematic review was conducted following a predefined protocol (unpublished). The Preferred Reporting Items for Systematic Reviews and Meta-Analyses (PRISMA) guidelines were followed in reporting.^19^

### Inclusion and exclusion criteria

This study focused on primary care, and included any primary care staff as participants. Training related to conducting remote consultation, including telephone, text, video, or email communication where patients were examined.

Empirical studies of any design, including unpublished research, was included. Review articles, conference abstracts, discussion and commentary articles, and letters were excluded.

We included studies published in English from 2010 onwards, owing to low level of remote consultation use with patients in primary care before this date. Studies that were conducted in non-primary care settings, or those that focused on student training, were excluded.

### Outcome measures

The review explored models of training and the content of curricula. Outcomes relating to training (type, provider, participants, completion rates), effects on healthcare professionals (impact on confidence, practice, satisfaction with training) and health service-related outcomes (impact on levels of remote consulting) were examined.

### Information sources

The following electronic databases were searched in December 2021: Medline (Ovid), Cochrane Database, PubMed, Embase (Ovid), Web of Science, and CINAHL. Additionally, Google was used to search for eligible published and unpublished studies in June 2022. Reference sections and citations of included studies were screened to identify further eligible studies.

### Search strategy

Search terms for the main search strategy included all terms relating to ‘remote consultation,’ ‘training’, and ‘primary care’. The full search strategy can be found in Supplementary Box S1.

We searched the first 100 results in Google for studies using a combination of terms. Search one was for the terms ‘general practice’, ‘remote consultation’, and ‘training’. Search two was for the terms ‘primary care, ‘remote consultation’, and ‘training’.

### Data management and screening

Search results were combined and duplicates eliminated using Endnote X9 and Covidence software. Eligibility of studies was assessed by screening titles and abstracts. Studies that met the inclusion criteria were further screened by full text by two researchers. In case of discrepancies, a third reviewer was consulted. Relevant data were collected using a data extraction template.

### Outcomes

The outcomes of interest were training (type, provider, participants, completion rates), effects on healthcare professionals (impact on confidence, practice, satisfaction with training) and health service-related outcomes (impact on levels of remote consulting).

### Quality assessment

We assessed quality of the included studies using the Mixed Methods Appraisal Tool (MMAT) 2018,^20^ appropriate for studies using a range of methodologies. Each study was assessed using five assessment points, and given an overall quality rating (for contextual purposes) based on the number of positive or negative scores received. Studies achieved an overall rating of high quality if four or five criteria were met, moderate quality if three criteria were met, and low quality if two or fewer criteria were met.^21^

### Data analysis

Included studies were analysed using narrative synthesis owing to their heterogeneous nature, enabling those with different designs to be analysed systematically, with similarities and differences being considered.^22^

Details of included studies and analysed outcome data were grouped according to characteristics and, where possible, into themes. Findings are presented using text and tables, with results of the quality assessment presented alongside to contextualise the synthesis.

## Results

In total, 1382 results were screened, resulting in the inclusion of ten studies in the review. The screening process and numbers and reason for exclusions can be found in the PRISMA flowchart (Supplementary Figure S1).

Of the ten studies included in the review, one was published before 2019,^23^ two in 2019,^24,25^ four in 2020,^26–29^ and three in 2021.^30–32^ One study was conducted in The Netherlands,^32^ three in the UK (specifically England),^24–26^ and six in the US.^23,27–31^ The most common study design was quantitative, including seven surveys and pre-intervention and post-intervention designs^24,25,27,29–32^ and one randomised controlled trial.^23^ One study was qualitative^28^ (involving case studies) and one was mixed-methods (involving questionnaires and interviews;^26^ Table 1).

**Table 1.** Characteristics of included studies

Author, year	Country of study	Setting and participants	Study design
Beaney, 2019^24^	England	General practice in Staffordshire; 24 general practice nurses from 19 practices	Quantitative design using survey. Pre-involvement, immediate post-involvement, and 2 months post-involvement surveys on competence/confidence/knowledge. Reflection on individual action plans for implementing TEC, including video consulting. LCAV questionnaires.
Chambers, 2019^25^	England	General practice in Staffordshire; 40 local general nurse practitioners	Quantitative design. Mix of feedback after workshops/learning conference of personal experiences. Individual practice reports from CCG with relevant TEC, including video consulting, to support outcomes. Use of TEC 6 months after programme.
Chaudhry, 2020^26^	England	General practice in London; GP trainees in NCEL London at any stage in their specialist GP training at one of the NCEL vocational training schemes	Mixed-methods design. Mainly quantitative using questionnaire. Also used semi-structured interviews. Reviewed experiences of GP trainees with remote consultation.
Jenkins, 2020^27^	US	Family medicine residency programme; residents in second year of training	Quantitative design using survey before undertaking curriculum, survey after undertaking curriculum. Patient evaluation forms during consultation.
Keyserling, 2021^30^	US	Internal medicine residents in continuity clinic; 16 first-year internal residents	Quantitative design using survey before training, and 1 week and 3 months after completing course.
Kirkland, 2021^31^	US	100 internal medicine residents per year (2016–19) at the Medical University of South Carolina	Quantitative design using pre-test assessment before didactic module, and a post-test and self-assessment evaluation after completion of the training.
Lawrence, 2020^28^	US	Primary care internal medicine residents at the New York University Grossman School of Medicine	Qualitative study using case study.
Paladine, 2010^23^	US	Family medicine residents from 16 residency programmes	Randomised controlled trial. Pre-intervention and post-intervention questionnaires, with residency programmes randomised into intervention or control groups.
Van Houwelingen, 2021^32^	Netherlands	Nurses (who had already used telehealth) employed in primary care, homecare, or hospital care in The Netherlands; 37 nurses across three teams.	Quantitative design using pre-test, post-test method during a tailored nursing telehealth training programme in homecare, primary care, and a hospital setting.
Wong, 2020^29^	US	56 first and third-year internal medicine residents in ambulatory block at a university-based residency programme in Stony Brook, New York (each session had 10–12 participants)	Quantitative design using survey before first session, after first session, and after second session.

CCG = clinical commissioning group. LCAV = Leading Change, Adding Value. NCEL = North Central and East London. TEC = technology enabled care.

Seven studies examined training on remote consultation for trainee GPs or residents,^23,26–31^ while three examined training of fully qualified primary care nurses.^24,25,32^ Participants were at varying stages in their careers and presented a heterogenous sample for analysis.

### Quality assessment

Four studies were rated as high quality,^24,30–32^ five were rated as moderate quality,^23,26–29^ and one was rated as low quality.^25^ The most common domain not achieved by included studies was whether participants were representative of the target population,^24,27,29–31^ rated as ‘cannot tell’ in seven quantitative studies (see Supplementary Table S1).

### Training style

The articles presented a range of training styles. Six studies^24,25,27,29–31^ collected data after implementing workshop or didactic learning followed by experiential learning, using either real or simulated patients. In contrast, two studies^23,32^ involved training with no practical elements attached, and the remaining two^26,28^ collected data either after an assessment of a remote consultation (as a workplace-based assessment or observed structured clinical examination [OSCE]) or after experiential learning without any previous teaching. Not all articles clearly stated whether their training, particularly with didactic elements, was online or in-person, and there were no consistent training methods between them (see Supplementary Table S2).

### Impact on healthcare professionals

All ten included studies report outcomes relating to healthcare professionals following training for remote consultation. Six studies reported increased confidence and self-efficacy when delivering consultation remotely,^23,24,27,30,31,33^ including improved knowledge of technology use.^23,33^ Participants reported positive responses about training on remote consulting across all studies; however, some highlighted that further training was probably needed.^27,29^

There was a heterogenous sample of trainees across and within articles, but many focused on preparedness for independent practice and confidence in remote methods. Only one article^26^ explored UK GP trainees’ experiences of training in remote consulting, solely telephone consulting, and highlighted the experiential nature of current training with assessment primarily through workplace-based assessments. Senior trainees reported more positive experiences with remote consulting, but all agreed that further training was needed.

The US studies employed a wide range of educational methods across diverse trainee groups and differing consultation modalities. Two articles did not state which year groups were examined,^23,28^ and one compared OSCE outcomes between postgraduate year 1 and 3 trainees after training (no discernible differences were identified).^29^ There was a wide range of remote consulting modalities trained for, with one study focusing on email only,^23^ and the rest a mixture of synchronous and asynchronous consulting. Despite the heterogeneity across studies, many reported that training increased appreciation for remote consulting, and trainees could use telehealth in their future independent practice.^27–29,31^ In one study, residents’ rated competence in remote consulting increased from 2% (*n* = 2/89) to 41% (*n* = 24/58) after training.^31^ Many studies, however, reported a need for further training.

Three studies explored outcomes for a mixed sample of primary care nurses across varying consultation modalities.^24,25,32^ Two UK-based studies^24,25^ evaluated outcomes for nurses in band 6 and advanced practitioner roles, but did not differentiate between the groups, with one focusing on consulting with patients with respiratory conditions.^25^ Across all three studies, training focused on electronic written communication with patients, either through a web-based platform or an app, with the addition of video consulting in the UK articles.

Training improved reported knowledge of remote consulting, with median knowledge rated immediately after training increasing from 2.9 to 3.7 out of 5.^32^ Nurses reported that training in, and subsequent use of, remote consulting was beneficial for use with patients and improved care.^24,25^

### Type of training

The studies assessed a wide range of training and pedagogical methods, including didactic teaching, workshops, and online learning. However, there was inconsistent delivery and lack of clarity within the studies. Two studies referenced online learning, one lecture-based learning, and the other ‘modules’ with associated discussion forums.^23,31^ Other studies described ‘didactic small group sessions’ but lacked details on their delivery.^27,30,32^ Additionally, the amount of teaching varied from one-off sessions to a 3-year curriculum, and experiential learning ranged from simulation to real patients at different stages of training.

Studies that implemented workshops or didactic learning alongside experiential learning reported more positivity and confidence in using remote consulting.^24,25,27,29–31^ Those studies that employed only one type of training (that is, lecture-based or experiential) particularly highlighted knowledge gaps and a desire for further training.^26,28,29^ In one study, after receiving lecture-based training, more participants stated they were ‘not sure’ about their comfort levels in using email communication with patients (0–17.5%, *P* = 0.003).^23^

### Impact on health services

Four studies reported health service outcomes related to the use and impact of remote consultation. Of these, three reported increased use of remote consultation and technology to deliver healthcare post-training,^24,27,32^ with one study reporting 89% (*n* = 17/19) of practices surveyed using three or more remote services.^24^

Studies involving fully qualified staff reported that participants felt training on telehealth or remote consulting and subsequent integration into practice would increase efficiency. Nurse participants in two studies^24,25^ reported increased clinician productivity, improved communication with patients, and fewer missed appointments after training. In one study, the number of remote consultations carried out by the nurse participants increased from 2 to 12.^32^

## Discussion

### Summary

Training in use of remote consultation led to increased confidence and skill in using remote methods, and was considered as a positive exercise among participants. It appears from the included studies that more intensive training results in more learning; however, there are limited studies varying in sample and design. These findings are in keeping with educational literature, where additional experiential experience enables the demonstration of higher-level learning and thinking.^34,35^

While most studies focused on providing training to trainee clinicians, fully qualified staff also reported improvements in clinical and patient outcomes, although many still identified gaps. This suggests that there is relevance and importance in training both populations to deliver consultation remotely.

Eight of the studies were conducted pre-pandemic, before primary care was forced to rapidly implement a wholly remote approach to consultation delivery. This increase in use has only reinforced the need for more evidence on training needs of primary care staff on remote consultation.

### Strengths and limitations

This review is necessary to assist with providing remote consultation in general practice, and includes up-to-date evidence, including information gathered during the COVID-19 pandemic.

The review only included studies published in the English language. This may have led to missing relevant evidence published in other languages. It is also possible that searches may have failed to identify studies that are poorly indexed or traverse disciplines.

The included studies were inconsistent in their approach, methodology, participants, settings, and aims, reflecting the novelty of this field, but making summary and comparison of training difficult. Some bias can occur when comparing the effects of training with no training, as changes in behaviour may occur due to the awareness of being observed.

This review aimed to examine models of training and the content of curricula, but limited published evidence that included these details was identified.

### Comparison with existing literature

A recent Cochrane review highlighted the need to train healthcare professionals in remote consulting.^13^ While it was uncertain as to whether a training intervention would improve telephone consulting skills, the review only included one study.

Studies show that clinicians’ confidence levels can vary when conducting remote consultation, but with proper training, their confidence can improve, as seen in this review.^3^ There has been hesitancy to integrate training into practice owing to a lack of perceived benefits, especially before the COVID-19 pandemic.^11^ However, this review has identified studies that demonstrate the positive outcomes of training in, and integration of, remote methods, such as increased work efficiency and better patient engagement.

These views are not limited to primary care; recent articles have recommended integrating remote consulting into dermatology and neurology training, and suggest options including supervised clinics, training days, and standardised guidance.^16,17^ Further evidence is needed to identify the best educational methods for this training, integrating the body of work with medical undergraduates in primary and secondary care settings.^15,33^ While postgraduate training needs may differ, these studies highlight the potential methods for, and benefits of, training in remote consulting.

### Implications for research and practice

Out of the studies conducted, only one examined the possibility of training non-clinical primary care staff members, with most of the studies focused on training GP trainees and nurses. It is crucial to further explore the training needs and effects of training for remote consultation among primary care staff members.

The recent surge in research indicates a rising demand for evidence on how primary care can effectively provide remote consultation. Staff members require training in this area, with the level of training needed varying depending on the staff’s experience. Training methods such as workshops and hands-on learning have been shown to positively impact the confidence and perceived skill of recipients, and should be included as significant components in this type of training.

Studies on training for remote consultation for primary care staff are few. Therefore, we are a long way from the necessary evidence-based curriculum and learning outcomes for training suitable for different types and grades of staff. Given the urgency and the current state of general practice, this educational programme requires proactive funding.

## Data

The dataset relied on in this article is available from the corresponding author on request.
